# Thiazolidinediones versus metformin on improving abnormal liver enzymes in patients with type 2 diabetes mellitus: a meta-analysis

**DOI:** 10.18632/oncotarget.24222

**Published:** 2018-01-13

**Authors:** Chunmei Xu, Junyu Zhao, Xiaojun Zhou, Rui Zhang, Tianyue Xie, Zhiwei Zou, Lin Liao, Jianjun Dong

**Affiliations:** ^1^ Department of Endocrinology, Shandong Provincial Qianfoshan Hospital, Shandong University, Jinan, China; ^2^ Department of Medicine, Division of Endocrinology, Qilu Hospital of Shandong University, Shandong University, Jinan, China; ^3^ Department of Endocrinology, Shandong Provincial Qianfoshan Hospital, Shandong University of Traditional Chinese Medicine, Jinan, China

**Keywords:** liver enzyme abnormalities, thiazolidinediones, metformin, randomized controlled trials, meta-analysis

## Abstract

**Background:**

Liver enzyme abnormalities are common in patients with type 2 diabetes. Currently, the inverse relationship between elevated liver enzymes and antidiabetics intake may be explained by rigorous treatment and good control. However, few studies have directly explored the influence of antidiabetics on abnormal liver function, especially the comparison between two insulin sensitizers—thiazolidinediones and metformin.

**Materials And Methods:**

Databases, including PubMed, Cochrane, CNKI, Wanfang and VIP were searched. Two reviewers performed independently. Meta-analysis was used when studies were homogeneous enough.

**Results:**

Six studies, including 4726 patients with type 2 diabetes, were involved in this systematic review. Compared with metformin, thiazolidinediones significantly reduced the alanine transaminase, aspartate aminotransferase and gamma-glutamyl transpeptidase. Further subgroup analysis suggested that pioglitazone-treated participants showed vast improvement in decreasing alanine transaminase (MD = -13.70; 95% CI = -16.91 to -10.52; *P* < 0.00001; I^2^ = 1%), aspartate aminotransferase (MD = -3.51; 95% CI = -5.74 to –1.28; *P =* 0.002; I^2^ = 0%) and gamma-glutamyl transpeptidase (MD = -5.41; 95% CI = -9.40 to -1.42; *P =* 0.008; I^2^ = 0%), while rosiglitazone exhibited no difference in lowering corresponding liver enzyme levels. Besides, thiazolidinediones similarly decreased fasting plasma glucose. However, thiazolidinediones were inferior to metformin in lowering HbA1C and alkaline phosphatase. Additionally, no significant publication bias was seen.

**Conclusions:**

Thiazolidinediones may confer modest biological improvement of liver function in people with type 2 diabetes than metformin. But owing to the limited methodological quality, more clinical researches are warranted in the future.

## INTRODUCTION

Diabetes mellitus (DM), which is a common chronic disease giving rise to numerous complications, has aroused the public’s attention around the world. Currently, metformin has become the pharmacological cornerstone in the treatment for patients with type 2 diabetes mellitus (T2DM) [1]. When metformin does not suffice or is contra-indicated, the alternative oral treatment options are: sulphonylureas (SUs), a-glucosidase inhibitor, thiazolidinediones (TZDs), dipeptidyl peptidase-4 (DPP-4) inhibitors [2]. It has been shown that the prevalence of elevations of alanine transaminase (ALT) was 3 to 4 times higher in patients with T2DM than in patients without diabetes [3]. Furthermore, patients with DM and fatty liver are remarkably insensitive to insulin. Most of patients with fatty liver or non-alcoholic steatohepatitis (NASH) typically have mildly elevated aminotransferase enzyme levels which frequently oscillate in and out of the normal range. Thus for patients with T2DM, it is significant to perform pre-treatment liver tests in order to distinguish the reasons for later liver enzyme abnormalities as drug-induced liver damage or not [4]. Yet, currently there are limited established studies monitoring liver function among patients with T2DM applying oral antidiabetics and exploring the specific effects of these hypoglycemic agents on improving liver function. In addition, NASH is the main hepatic pathologic manifestation that concomitantly occurs to diabetics. Nowadays, no effective measures could cure NASH other than lifestyle modification and weight loss, which are often of trouble to achieve and even harder to maintain [5–7]. TZDs and metformin, as the two main groups of insulin-sensitizing drugs, may offer the therapeutic benefits for NASH treatment [8]. Despite of the widespread use of insulin-sensitizing drugs in clinical practice, a growing number of patients began to be concerned about the side effects of insulin sensitizers, including abnormality of renal function, and in particularly, liver damage. The purpose of this meta-analysis of randomized controlled trials is to summarize the currently available evidence for the effect of insulin-sensitizing agents on biochemical endpoints regarding both liver function and plasma glucose level in diabetics.

## MATERIALS AND METHODS

### Searching methods for identification of studies

A systematic search was undertaken by two independent reviewers through searching the following database: PubMed, Cochrane, CNKI, VIP and Wanfang data with disagreements resolved by consensus. There were various searching words for a variety of databases. The searching terms we used were related to interventions, disease and outcome indicators. Our searching strategy included as following: (Thiazolidinediones OR Thiazolidinedione OR TZDs OR rosiglitazone OR pioglitazone) AND (metformin OR glucophage) AND (Liver function OR Alanine Transaminase OR ALT OR Aspartate Aminotransferases OR AST OR gamma-Glutamyltransferase OR GGT OR Alkaline Phosphatase OR AKP) AND (RCT OR controlled clinical trial OR randomized). Databases were searched from the earliest data to 1 July 2017.

### Inclusion and excluded criteria

Two reviewers independently browsed through the titles and the abstracts identified according to the above-described strategy. And they reached consensus through consultation. If an agreement couldn’t be reached, a third reviewer would decide. All potentially relevant essays were retrieved, and the full text of these studies was read over to determine which trials satisfied the inclusion criteria. Inclusion criteria were randomized controlled clinical trials of patients with T2DM with abnormal liver function tests, who were treated with thiazolidinedione drugs whether with rosiglitazone or pioglitazone of any dose versus metformin of any dose, with or without other interventions or healthy lifestyle management such as diet control or exercise. Conversely, Non-RCT trials or/and trials of patients with non-T2DM or/and trials without TZDs and metformin for comparison or/and liver enzymes not mentioned were excluded.

### Data extraction

The two authors extracted the following data for all included trials independently. Items included were title, author, country, date, number of participants, inclusion and exclusion criteria, mean (or median) age, sex ratio, intervention and dose, follow-up length, duration of diabetes and/or of additional intervention(s), and outcome measures. The characteristics of the articles were recorded in a form.

### Methodological quality and risk of bias

We assessed the influence of methodological quality to avoid the risk of overestimation of intervention effects. The specific assessment methods were used via Cochrane definitions: generation of the allocation sequence (high or low or unclear risk); allocation concealment (high or low or unclear risk); blinding of participants and personnel (high or low or unclear risk); blinding of outcome assessment (high or low or unclear risk); incomplete outcome data (high or low or unclear risk); selective reporting (high or low or unclear risk). Two reviewers independently evaluated these items.

### Missing data

Where data were missing, corresponding data were extracted from other sources. On condition that data had not been reported sufficiently or were not published at all, we would correspond with the authors to acquired further information.

### Data analysis

The main outcomes were the change of biochemical parameters from baseline, including serum activities of alanine transaminase (ALT), aspartate aminotransferase (AST), gamma-glutamyl transpeptidase (GGT), alkaline phosphatase (AKP). Besides the efficacy of the hypoglycemic drugs on the liver, change of fasting plasma glucose (FPG) and glycosylated hemoglobin (HbA1C) [9] were also examined. The statistical package that RevMan5.3 and Stata version 12.0 (StataCorp, College Station, TX, USA). provided was used. For continuous variables, the mean difference (MD) with 95% interval was calculated. The fixed-effects model was applied to synthesize the data of the different trials when there were no significant heterogeneities; otherwise, the random-effects model was used [10]. Subgroup analysis by kind of TZDs (either rosiglitazone or pioglitazone) was applied to assess the efficacy of different therapeutic group. The I^2^ was calculated which can be interpreted as the percentage of the variation between studies that attributes to heterogeneity rather than chance. I^2^ = 0% indicates no heterogeneity, and I^2^ = 100% represents that all variation derives from heterogeneity. Sensitivity analyses excluding one study at a time were also performed to evaluate whether any specific study significantly influenced the overall pooled results. Publication bias was evaluated via Begg’s funnel plots [11]. Statistical levels of significance were estimated with *P* < 0.05.

## RESULTS

### Search results

**Figure 1:**
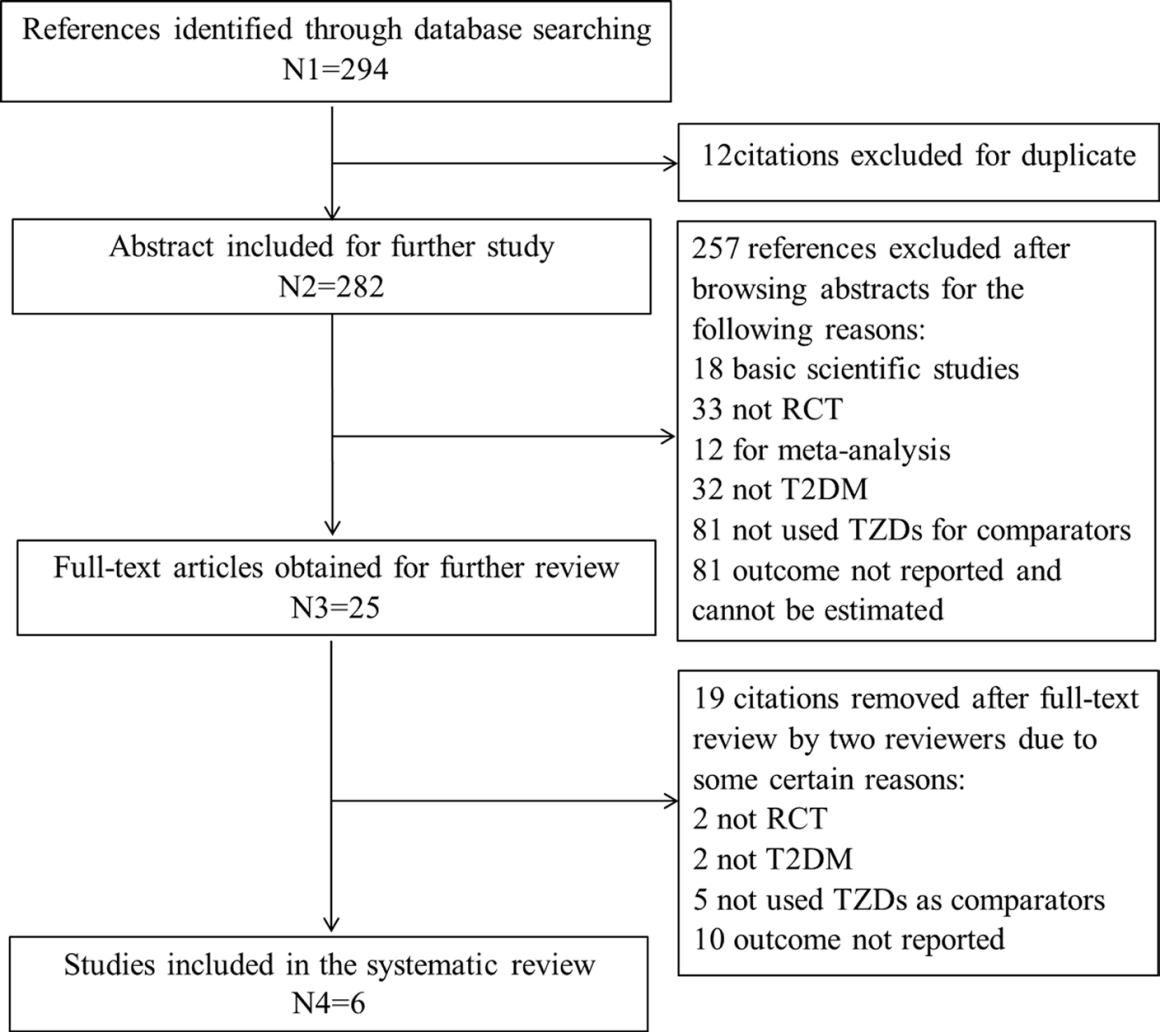
Literature search flow diagram

Our initial search identified 294 references from PubMed and Cochrane, and no reference gained from Chinese database according to the agreement for study selection. After getting rid of the duplicate, 282 references remained to be looked through the abstracts. Only 25 references were assessed for full-text review because of the failure of study design or intervention or disease or outcome measure. We excluded 19 references for the previous reasons, the remaining six references were considered for inclusion. And the searching progress was displayed in Figure 1.

### Study characteristics

Among the final six references, two studies were conducted in Europe [12–13], one in Greek [14], one in Japan [15], one in Turkey [16], and one in Europe, Canada, and Australia [17]. The main characteristics of the studies were shown in Supplementary Table 1. The six randomized controlled trials [12–17] were published as full text articles and included 4726 patients. The sample size of each arm varied apparently, ranging from 14 to 1857 participants. The experimental intervention was TZDs, and we chose rosiglitazone [14, 16] and pioglitazone [12, 13, 15, 17] as intervention measures, except for troglitazone due to the known hepatotoxicity [18, 19]. The trials we included contained not only metformin or TZDs monotherapy but also adding-on to SUs [12, 13]. Only in the T.Karo 2009 et al. trial, 50 patients received one-month exercise therapy including 50 min or more of exercise per week [15]. All except for two trials [12, 13] of the group, patients received diet. In the F.Iliadis 2007 et al. trial [14], all participants suffered from T2DM concomitantly with NASH. The durations of the intervention periods varied from 12 to 52 weeks.

### Methodological Quality

Of all studies, four studies mentioned the specific randomized method [12, 14–16], but the remaining two referred to “random” but no method in detail [13, 17]. Two studies adopted allocation concealment [15, 17], and five studies were double-blind [12–15, 17]. Apart from the T.Karo 2009 et al. trial [15], other five studies had incomplete outcome data bias. And the reporting bias was only mentioned in the G.Belcher 2004 et al. trial [13]. In brief, the methodological quality of the included studies we had them in this meta-analysis was not good.

### Outcomes

#### Liver function tests

#### ALT with monotherapy

**Figure 2:**
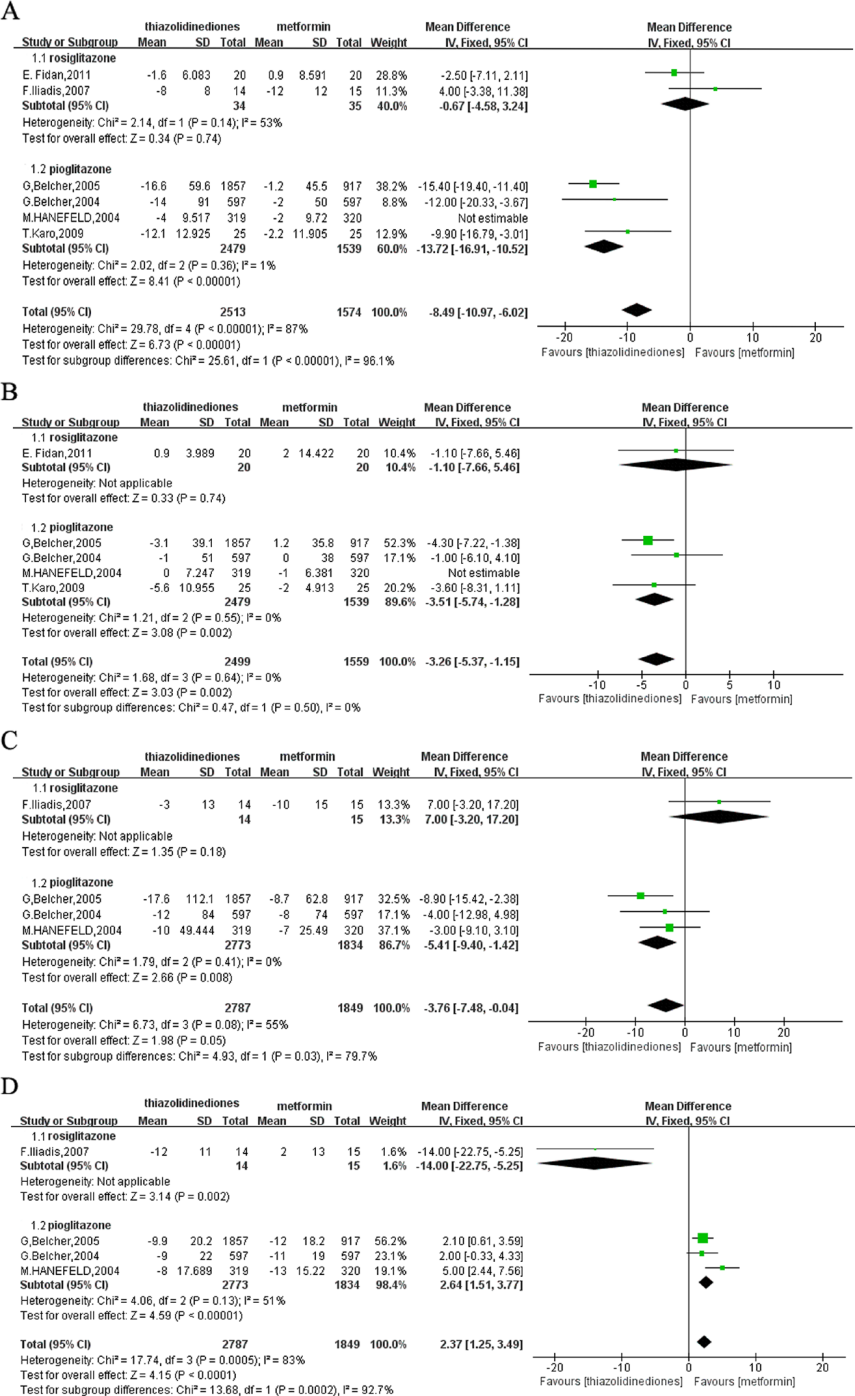
Forest plot of the improvement of liver enzymes from baseline to the end of treatment with monotherapy (**A**) ALT. (**B**) AST. (**C**) GGT. (**D**) AKP. ALT: alanine transaminase; AST: aspartate aminotransferase; GGT: gamma-glutamyl transpeptidase; AKP: alkaline phosphatase.

All of the six studies [12–17] were available to investigate the serum activities of ALT, and the total number of participants was 2832 and 1894 respectively for TZDs or metformin monotherapy. The pooled results of six RCTs revealed a significant difference with high heterogeneity on the change of serum ALT levels from baseline in patients treated with TZDs compared with metformin. Considering that different class of TZDs was applied by different studies, subgroup analysis of the fixed-effects model was conducted. Rosiglitazone group [14–16] displayed the moderate heterogeneity but no difference of the reduction of ALT (MD = 0.67; 95%CI = -4.58 to 3.24; *P* = 0.14; I^2^ = 53%). Due to substantial heterogeneity in the group treated with pioglitazone [12, 13, 15, 17], we conducted sensitivity analysis by excluding a study conducted by M.Hanefeld et al. [12] for adding-on to SUs as therapy. The result found a more significant decreased effect on ALT by pioglitazone than metformin (MD = -13.72; 95%CI = -16.91 to -10.52; *P* < 0.00001; I^2^ = 1%) (Figure 2A).

### AST with monotherapy

Another evaluating indicator of liver enzymes, AST, was assessed from included five trials [12, 13, 15–17]. Similarly, subgroup analysis of the fixed-effects model was conducted by grouping for the class of TZDs, and the result showed that there was no difference in the reduction of AST between the rosiglitazone group and metformin group with unmeasurable heterogeneity for only one study included [16] (MD = -1.10; 95% CI = -7.66 to 5.46; *P* = 0.74). However, pioglitazone was more effective than metformin in the reduction of AST (MD = -3.51; 95% CI = -5.74 to -1.28; *P* = 0.002; I^2^ = 0%) with no heterogeneity after excluding one trial that was responsible for heterogeneity for its adding-on to SUs intervention [12] (Figure 2B).

### GGT and AKP with monotherapy

Four studies [12–14, 17] measured the biochemical response of GGT and AKP. And the fixed-effects model was used to merge MD values and the pooled MD was -3.76 (95% CI: -7.48 to -0.04; *P* = 0.05) with moderate heterogeneity (*P* = 0.08; I^2^ = 55%) for GGT, which indicated that TZDs would decrease GGT more remarkablely than metformin. Further subgroup analysis result indicated that rosiglitazone had similar efficacy in reducing GGT level with metformin (MD = 7.00; 95%CI = -3.20 to 17.20; *P* = 0.18). By contrast, pioglitazone significantly decreased GGT compared with metformin (MD = -5.41; 95%CI = -9.40 to -1.42; *P* = 0.008; I^2^ = 0%) (Figure 2C). Due to the substantial heterogeneity from these studies (*P* = 0.0005; I^2^ = 83%), subgroup analysis of the fixed-effects model was used to merge MD values of AKP. The meta-analysis found that there was statistically significant reduction between participants treated with rosiglitazone and metformin (MD = -14.00; 95% CI = -22.75 to -5.25; *P* = 0.002) with undetected heterogeneity for only including one study. Besides, the pooled data for pioglitazone group was 2.64 (95% CI = 1.51 to 3.77; *P* < 0.00001) with moderate heterogeneity (*P* = 0.13; I^2^ = 51%), which indicated that pioglitazone was inferior to metformin in lowering AKP (Figure 2D).

### Adding-on to SUs

**Figure 3:**
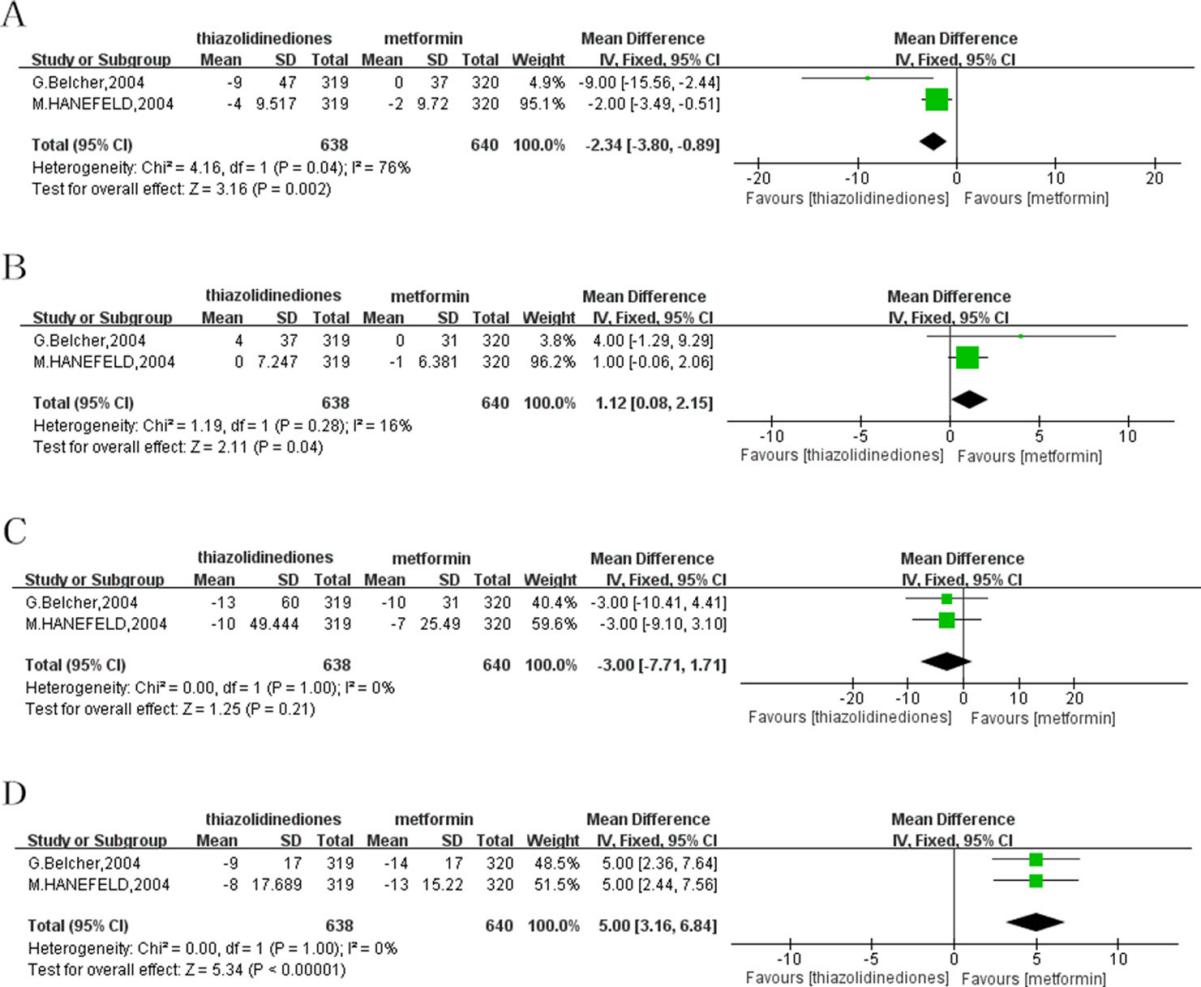
Forest plot of the improvement of liver enzymes from baseline to the end of treatment with adding-on to SUs (**A**) ALT. (**B**) AST. (**C**) GGT. (**D**) AKP. ALT: alanine transaminase; AST: aspartate aminotransferase; GGT: gamma-glutamyl transpeptidase; AKP: alkaline phosphatase; SUs: sulfonylureas.

Apart from monotherapy, treatment with adding-on to SUs was reported by two studies. One research which was excluded by above meta-analysis was conducted by M.Hanefeld et al. [12]. and only used combination therapy with adding-on to SUs as intervention. Another study performed by G.Belcher et al. [13]. applied with both monotherapy and combination treatment with SUs. Among the two studies adding-on to SUs, the decrease of ALT from baseline to the end of treatment was more significant when treated with TZDs in comparison with metformin with a MD of -2.34 (95% CI: -3.80 to -0.89; *P* = 0.002; I^2^ = 76%). As to the reduction of AST, the result showed that TZDs was inferior to the treatment of metformin with adding-on to SUs, and the pooled data was a MD of 1.12 (95% CI: 0.08 to 2.15; *P* = 0.04; I^2^ = 16%). However, there was no significant difference in GGT reduction with pioglitazone compared with metformin, with a MD of -3.00 (95% CI: -7.71 to 1.71; *P* = 0.21; I^2^ = 0%). Similar to the change of AST, analysis indicated that metformin with adding-on to SUs showed a more significant role in the reduction of AKP compared to TZDs (MD = 5.00; 95%CI: 3.16 to 6.84; *P* < 0.00001; I^2^ = 0%) (Figure 3).

### Efficacy on lowering glucose

#### FPG

**Figure 4:**
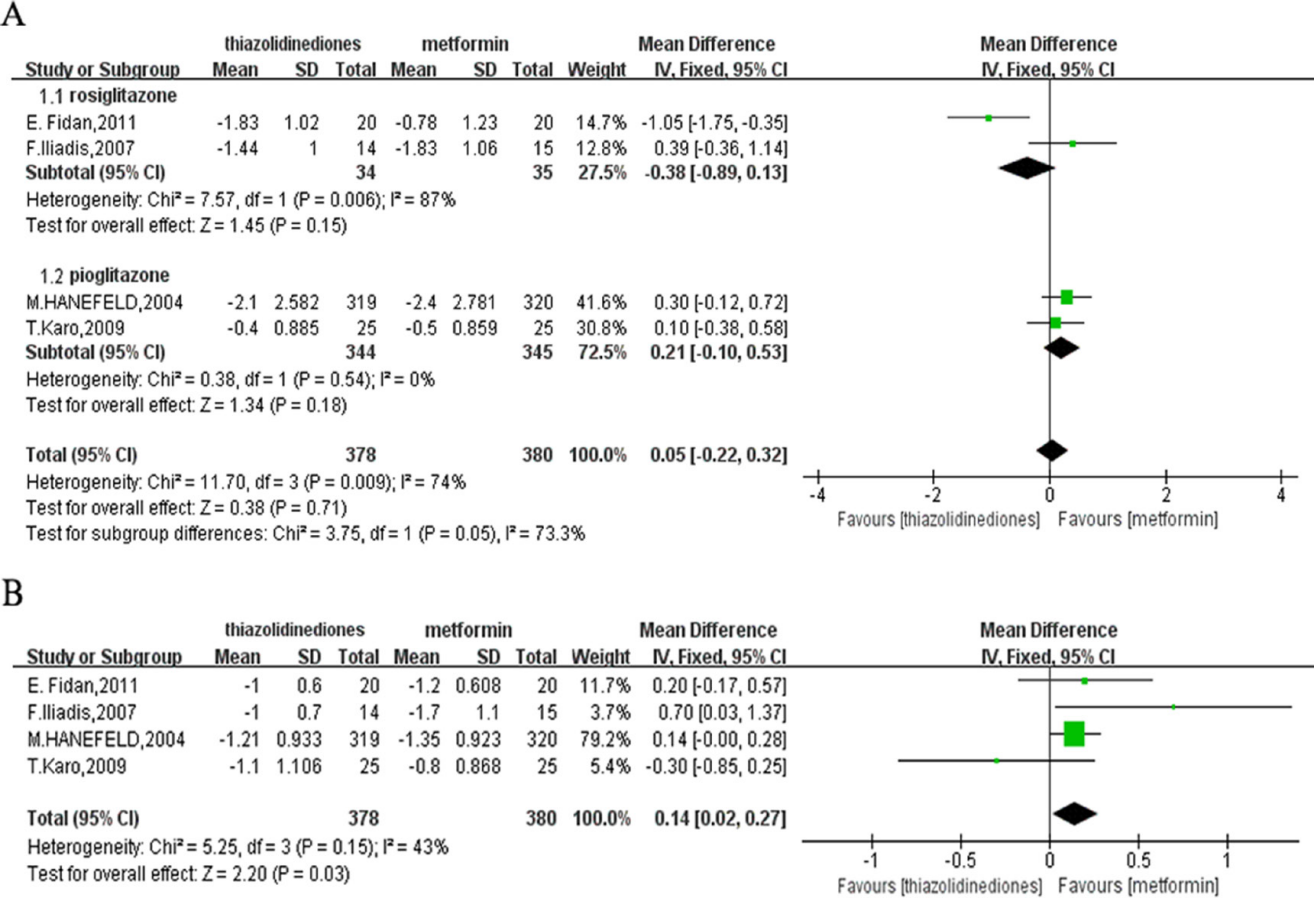
Forest plots comparing the effects of lowering glucose (**A**) FPG. (**B**) HbA1C. FPG: fasting plasma glucose; HbA1C: glycosylated hemoglobin.

Four studies [12, 14–16] contributed to the FPG analysis with a total of 758 participants, 378 and 380 in the TZDs and metformin group, respectively. There was no difference of the change of FPG from baseline to the end of treatment in four trials by calculating the MD (MD = 0.05; 95%CI: -0.22 to 0.32). However, this result was built around the premise that heterogeneity was higher we couldn’t neglect (*P* = 0.009; I^2^ = 74%). Hence, to eliminate the heterogeneity, the subgroup analysis was performed. For one group treated with rosiglitazone [14, 16], the heterogeneity still existed (*P* = 0.006; I^2^ = 87%) and the pooled MD was -0.38 (95% CI: –0.89 to 0.13; *P* = 0.15), which showed no difference between rosiglitazone and metformin. And for another group with pioglitazone [12–15, 17], the fixed-effects model was used to merge MD values of FPG and a similar result was found with the pooled MD was 0.21 (95% CI: –0.10 to 0.53; *P* = 0.18) with no heterogeneity (*P* = 0.54; I^2^ = 0%) (Figure 4A).

### HbA1C

The same four studies [12, 14–16] as reporting FPG were available to measure the level of HbA1C. Compared to the participants with TZDs, metformin was more effective with the pooled MD of 0.14 (95% CI: 0.02 to 0.27) with moderate heterogeneity (*P* = 0.15; I^2^ = 43%). Nevertheless, due to the reduction by 0.27 at most of HbA1C in the metformin group than TZDs group, the effective of metformin on HbA1C reduction might have little clinical significance (Figure 4B).

### Publication bias

**Figure 5:**
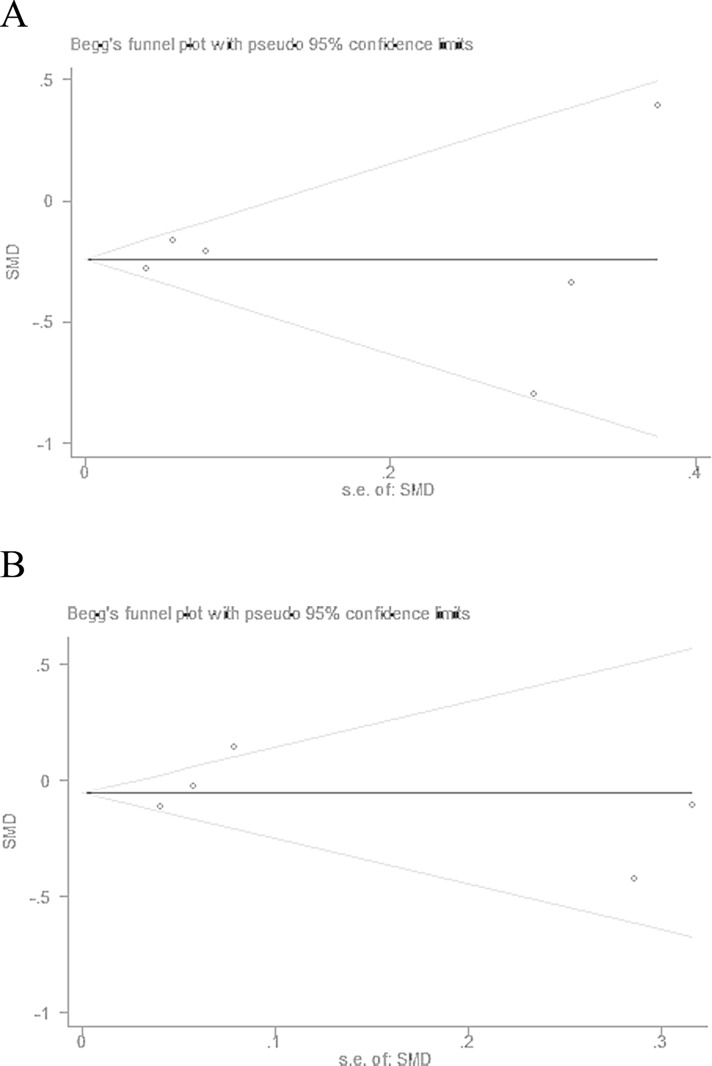
Funnel plots of ALT (**A**) and AST (**B**) revealed no significant publication bias. SMD: standardized mean difference; s.e. of SMD: standard error of standardized mean difference.

The funnel plots based on the ALT (Begg’s test: *P* = 0.707) and AST (Begg’s test: *P* = 0.806) are shown in Figures 5A and 5B, respectively. The funnel plot shapes were symmetrical, indicating no obvious reporting bias.

## DISCUSSION

Liver enzyme abnormalities are common in patients with T2DM [20]. It has been shown that the prevalence of elevations of ALT was 3–4 times higher in patients with T2DM than in patients without T2DM [3]. Thus, for insulin-resistant T2DM, it is crucial to perform pre-treatment liver tests in order to be able to interpret later liver enzyme abnormalities observed after the initiation of drug treatment [4]. Insulin resistance seems to play a central role of accumulation of triglycerides in the liver and initiation of the inflammatory cascade. That is why insulin sensitizers can be regarded as beneficial treatment at this stage of liver injury [21]. As the top two representative drugs of insulin-sensitizers, TZDs and metformin are widely applied in clinics for diabetes [22–24]. It is worth noting that the first-generation TZD, troglitazone, the first compound approved by the Food and Drug Administration in the US, proved to be hepatotoxic and was withdrawn from the market after the report of several dozen deaths or cases of severe hepatic failure requiring liver transplantation [4]. However, the second-generation thioglitazones, that is, rosiglitazone and pioglitazone appear to be safer, although their use is currently contraindicated in the presence of active liver disease or of ALT more than 2.5 times normal [25]. Nowadays, there is no published study comparing the efficacy of the two insulin-sensitizers on the liver function.

Earlier systematic reviews have shown improvements across the histological and biological response of the liver treated with hypoglycemic drugs, while these researches studied people who suffered from NASH or NAFLD, not with T2DM. A systematic review performed by Suzanne E. et al. indicated that TZD treatment may be less effective in non-diabetics with NASH [26]. Nevertheless, an open-label, randomized, a single-center study revealed that rosiglitazone therapy seemed to be more effective in metabolic control and histological improvement in NAFLD patients with impaired glucose metabolism and metformin therapy alone has not improve serum transaminase levels [27]. In addition, another clinical trial showed that insulin sensitizers could lead to an improvement in metabolic, biochemical and histological parameters in NASH as a result of improved insulin sensitivity [28].

Since increased transaminase activity is an indicator of hepatic necroinflammation which could lead to fibrosis and cirrhosis of liver [29], transaminase level was regarded as an important outcome for monitoring liver function. Our results indicated that the level of ALT, a useful marker for measuring hepatocellular damage and reflecting cell membrane function with the highest concentration found in liver [30], was significantly reduced for the patients treated with TZDs than that of metformin group, especially treated with pioglitazone. Similar conclusions that long-term therapy with pioglitazone may be necessary to maintain improvements in serum aminotransferase in patients with NASH, while improvements in serum aminotransferase levels treated with metformin may not be sustained for even 48 weeks were reached in several studies [22, 25, 31]. Similar with ALT, there was statistically significant difference in the change of AST between treatment and control group. ALT and AST levels, measure the concentration of intracellular hepatic enzymes that have leaked into the circulation and serve as a marker of hepatocyte injury [32]. Some potential explanations for elevated transaminases in insulin-resistant states include oxidant stress from reactive lipid peroxidation and recruited inflammatory cells. And the insulin-resistant state is also characterized by an increase in proinflammatory cytokines such as tumor necrosis factor-α (TNF-α), which may also contribute to hepatocellular injury [32]. Indeed, a few possible mechanisms have been explored to explain the hepato-protective effects of TZDs, including amelioration of insulin resistance, reducing the TNF-α production, increasing adiponectin concentration, activation of AMP-related protein kinase and inactivation of the intracellular pro-inflammatory signaling pathway [33–36]. Besides, the ability to improve liver enzymes of TZDs may be also explained by activation of PPAR-γ, then causing downregulation of inflammation and fibrosis through its effect on Kupffer and hepatic stellate cells [27, 37].

As for the other markers for liver function tests, applying TZDs would decrease GGT more remarkablely, while metformin showed stronger efficacy in lowering AKP. Increased AKP mainly indicates the pathological changes in biliary flow, and highly concentrated bilirubin in serum causing damage to hepatocytes [38]. In contrast to metformin group, weaker improvement of AKP level for TZDs group may manifest less remission, which coincided with the finding of increased AKP levels for rosiglitazone-treated Akita animals after cholestasis liver injury [39]. Apart from the use of TZDs or metformin alone, the relevant results of combination with SUs resembled with the monotherapy. It revealed the significant decrease of ALT and similar capabilities in lowering GTT between TZDs and metformin, and stronger effect on the reduction of AST and AKP when combining SUs with metformin. Evidence has emerged that the hepato-protective impact of gliclazide was more prominent in patients with chronic liver disease. And one possible explanation was due to the innate characteristics of gliclazide, which is known as a free-radical scavenger [40]. Thus it could be seen that SUs would play a supporting role in the effect on the liver function.

Due to differences in their *in vivo* metabolic pathways and affinity of binding with PPAR-γ, rosiglitazone and pioglitazone demonstrate shorter accumulation than troglitazone, thereby reducing their relative hepatotoxicity [19]. However, the limited number of randomized trials available does not allow us to draw any definitive conclusion about the different efficacy of the two TZDs on improvement of abnormal liver enzymes, especially with limited research choosing rosiglitazone as intervention.

In terms of glucose-lowering effect, our study revealed that metformin lowered HbA1C more significantly than TZDs, while both insulin-sensitizers showed the similar effect on reducing FPG. Greater reduction in HbA1C indicated greater improvement in glycemic control. Metformin acting as the first-line hypoglycemic drug improves hepatic insulin resistance mainly by decreasing hepatic expression of TNF-α, a cytokine that promotes insulin resistance [25, 27, 41], ameliorates hepatic steatosis and decreases aminotransferase levels, thus causes reversal of fatty liver [27, 41]. Besides, beneficial action of metformin is thought to reduce excessive rates of hepatic glucose production. Moreover, glucose utilization by extra-hepatic tissues may also be enhanced through activation of AMPK [42]. However, TZDs enhance insulin sensitivity through stimulation of PPAR-γ, mainly acting in the skeletal muscles and liver as well as promoting adipogenesis of insulin-sensitive adipocytes [24]. Whereas, the only reduction by 0.27 at most of HbA1C in the metformin group had little clinical significance. From the above, the efficacy on the reduction of plasma glucose may be equal between two insulin-sensitizers.

Nevertheless, our results should not be overinterpreted. We cannot ignore the fact that there was considerable heterogeneity among six trials in respect to sample size, type of experimental interventions, the duration of interventions, and the drug doses. Besides, there was a considerable discrepancy in the methodological quality across the included trials. Only one trial was regarded as being of high methodological quality [15]. Thus more researches on this field needs to be done further. And further prospective clinical studies are warranted to increase our understanding of the relationship between insulin-sensitizers and improvement of abnormal liver enzymes.

## CONCLUSIONS

In conclusion, the findings of this systematic review suggest that compared with metformin, TZDs may confer modest biological improvement of liver function in people with T2DM. More future researches need to focus on the specific factors related to the change of liver enzymes, such as improvement of hepatic insulin resistance. Moreover, we will take the morphologic features of liver into account in further studies.

## SUPPLEMENTARY MATERIALS TABLE


