# Supplementary material for: Thiazolidinediones versus metformin on improving abnormal liver enzymes in patients with type 2 diabetes mellitus: a meta-analysis
Source: Oncotarget. 2018 Jan 13;9(15):12389–99. doi: 10.18632/oncotarget.24222 (PMC5844755; doi:10.18632/oncotarget.24222)
Supplement: Supplementary file 1 [file oncotarget-09-12389-s001.pdf]

Thiazolidinediones versus metformin on improving abnormal liver enzymes in patients with type 2 diabetes mellitus: a meta-analysis

SUPPLEMENTARY MATERIALS

Supplementary Table 1: Study characteristics for all randomized controlled trials in the systemic review

Primary study	Country	Disease	Diabetes Duration (years)		Number (*n*)		Age (years)		Male (%)		Intervention		Dosage (mg/d)		Add on to	Follow-up (weeks)
			Con	Tre	Con	Tre	Con	Tre	Con	Tre	Con	Tre	Con	Tre		
M.Hanefeld, 2004	Europe	T2DM	7.1 ± 5.6	7.0 ± 5.6	320	319	60 ± 8.0	60 ± 8.8	54.7	53.6	Met	Pio	2550	45	SUs	52
G.Belcher, 2004	Europe	T2DM	4.1 ± 4.7	4.2 ± 4.8	597	597	57 ± 9	57 ± 9	57	55	Met	Pio	2550	45	SUs	52
F.Iliadis, 2007	Greek	T2DM, NASH	1.7 ± 2.7	2.6 ± 2.6	15	14	57.8 ± 9.1	56.3 ± 12.8	17		Met	Ros	1700	8	No	18
T.Karo, 2009	Japan	T2DM, metabolic syndrome	Not available	Not available	25	25	58.6 ± 12.4	51.4 ± 15.2	56	48	Met	Pio	500	15	No	12
E. Fidan, 2011	Turkey	T2DM	Not available	Not available	20	20	52.6 ± 7.2	54.1 ± 9.0	70	55	Met	Ros	850–550	4–8	No	12
G.Belcher, 2005	Europe Canada Australia	T2DM	4.4 ± 4.9	4.2 ± 4.8	917	1857	58 ± 9.1	57 ± 9.4	56	55	Met	Pio	2550	45	No	52

a: Con: Control; b: Tre: Treatment; c: Met: Metformin; d: Pio: Pioglitazone; e: Ros: Rosiglitazone; f: SUs: Sulfonylureas; Data are *n* (%) and means± SD.
